# Phylogenetic Information Content of Copepoda Ribosomal DNA Repeat Units: ITS1 and ITS2 Impact

**DOI:** 10.1155/2014/926342

**Published:** 2014-08-18

**Authors:** Maxim V. Zagoskin, Valentina I. Lazareva, Andrey K. Grishanin, Dmitry V. Mukha

**Affiliations:** ^1^Vavilov Institute of General Genetics, Russian Academy of Sciences, Gubkin Street. 3, Moscow 119991, Russia; ^2^Papanin Institute for Biology of Inland Waters, Russian Academy of Sciences, Borok 152742, Russia; ^3^Dubna International University for Nature, Society and Man, Universitetskaya Street 19, Dubna 141980, Russia

## Abstract

The utility of various regions of the ribosomal repeat unit for phylogenetic analysis was examined in 16 species representing four families, nine genera, and two orders of the subclass Copepoda (Crustacea). Fragments approximately 2000 bp in length containing the ribosomal DNA (rDNA) 18S and 28S gene fragments, the 5.8S gene, and the internal transcribed spacer regions I and II (ITS1 and ITS2) were amplified and analyzed. The DAMBE (Data Analysis in Molecular Biology and Evolution) software was used to analyze the saturation of nucleotide substitutions; this test revealed the suitability of both the 28S gene fragment and the ITS1/ITS2 rDNA regions for the reconstruction of phylogenetic trees. Distance (minimum evolution) and probabilistic (maximum likelihood, Bayesian) analyses of the data revealed that the 28S rDNA and the ITS1 and ITS2 regions are informative markers for inferring phylogenetic relationships among families of copepods and within the Cyclopidae family and associated genera. Split-graph analysis of concatenated ITS1/ITS2 rDNA regions of cyclopoid copepods suggested that the *Mesocyclops*, *Thermocyclops,* and *Macrocyclops* genera share complex evolutionary relationships. This study revealed that the ITS1 and ITS2 regions potentially represent different phylogenetic signals.

## 1. Introduction

Copepods are important components of zooplankton and the food chain in marine and freshwater ecosystems. The subclass Copepoda is believed to contain approximately 13,000 morphospecies; however, the actual number of species in this subclass might be much greater [1]. The majority of the freshwater copepod species belong to the order Cyclopoida, which includes the free-living species (approximately 800) in the family Cyclopidae. The other two free-living families (Oithonidae and Cyclopinidae) contain mainly marine species except for a few species in Oithonidae [2].

Systematic analyses of cyclopoid copepods (order Cyclopoida) have primarily focused on morphological characteristics [2–8], and the majority of molecular studies have targeted marine copepods [9–41]. The phylogenetic history of freshwater cyclopoid copepods is not well understood. A few studies on Cyclopidae have used molecular and morphological analyses on the* Mesocyclops* genus (Crustacea: Cyclopidae) [42],* E. serrulatus* group [43],* Acanthocyclops vernalis-robustus* species complex [44, 45], and 11 populations of* Macrocyclops albidus* [46]; other phylogenetic analyses have focused only on molecular markers in* Diacyclops* spp., which are found in western Australia [47] and Lake Baikal [48].

Molecular markers such as genomic DNA fragments are used for phylogenetic analyses to elucidate the evolutionary history of living organisms, and the region of genomic DNA analyzed is critical. Mitochondrial DNA fragments (genes encoding the cytochrome c oxidase subunit I (COI), 16S rRNA, and cytochrome b) [9–21, 39, 40, 49–52] and/or nuclear rDNA regions have been used for the phylogenetic analysis of cyclopoid copepods [22–37, 41, 53–58]. Mitochondrial DNA fragments might be less useful for the analysis of copepod phylogeny compared to the phylogeny of other taxa; furthermore, amplification of COI is difficult in some copepods [59–61]. However, these DNA fragments might be informative for analyses of population differentiation or cryptic speciation [9, 11–15, 38–40, 50, 52]. Therefore, comparison of nuclear rDNA regions might be informative for the phylogenetic analysis of copepods.

**Figure 1:**
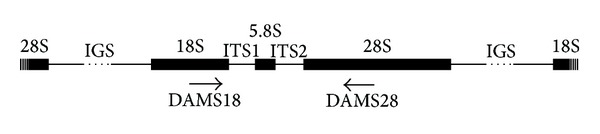
Organization of eukaryotic tandemly repeated rDNA clusters. 18S, 5.8S, and 28S ribosomal RNA genes; ITS1 and ITS2 internal transcribed spacers; IGS intergenic spacer. Arrows indicate the locations of the DAMS18 and DAMS28 primers.

In most eukaryotes, rRNA genes are located in a multigene family of genomic clusters of repeated sequences. Within these clusters, the 18S, 5.8S, and 28S rRNA genes are separated by internal transcribed spacers (ITS1 and ITS2) and an intergenic spacer [62] (Figure 1). Ribosomal DNA (rDNA) is a reliable and informative phylogenetic marker [63] that contains sequences with different rates of evolutionary variability. In most eukaryotes, the most evolutionarily conserved genes are the rRNA genes; comparison of their sequences allows estimation of the evolutionary distances at intergeneric and higher taxonomic levels. Comparison of the more evolutionarily variable spacer sequences enables the study of phylogenetic relationships at the species and population levels [63–65]. Therefore, comparison of different regions of rDNA enables the phylogenetic analysis of organisms over extended evolutionary distances.

Phylogenetic relationships among cyclopoid copepods at higher systematic levels (ordinal, familial, and generic) have been resolved using the 18S and 28S nuclear rRNA genes [28, 29, 55–57], and the relationships at the lower taxonomic levels (species and populations) have been resolved using the ITS2 of the nuclear rDNA gene cluster [30, 40, 42, 52, 58].

Notably, analysis of the evolutionary history of living organisms based on only one molecular marker can uncover bifurcating phylogenetic trees, revealing branched evolution. However, it recently becomes evident that evolution is not always tree-like. Comparisons of gene trees based on different genetic loci often reveal conflicting tree topologies. These discrepancies are not always due to the problems with the sampling and the gene tree reconstruction methods. Reticulation events such as horizontal gene transfer (HGT) and hybridization may be responsible for contradictions in lineages. During an HGT event, a DNA segment is transferred from one organism to another which is not its offspring, whereas hybridization describes the origin of a new species through an interspecies mating. Both processes yield genomes that are mixtures of DNA regions derived from different species. Consequently, evolutionary relationships between species whose past includes reticulation can often be better represented by using phylogenetic networks rather than trees [65–67].

In view of the above, comparing phylogenetic trees based on different molecular markers may be used for the analysis of evolutionary events caused by reticulate evolution. Phylogenetic signals from various molecular markers are potentially divergent during reticulate evolution, resulting in phylogenetic trees with alternative positions for the individual branches [68–71]. Comparative analysis of the rDNA ITS1 and ITS2 sequences is suitable for studying phylogenetic relationships in terms of branching and reticulate evolution [63, 68, 70, 72–82].

Reticulate evolution is primarily driven by hybrid speciation, which is common among plants [83] but also occurs among animals, particularly including fish [84], amphibians, and several invertebrates [85–87]. In both mammals [88] and arthropods [89, 90], a single instance of hybrid speciation has been well described. Interspecies hybridization typically results in complicated relationships within species complexes, characterized by indistinct species borders. Reticulate evolution among crustaceans has been observed only within species complexes of daphnids [91–94].

In this study, we analyzed the phylogenetic relationships within a small group of cyclopoid copepods representing several genera of freshwater (*Cyclops*,* Thermocyclops*,* Diacyclops*,* Megacyclops*,* Macrocyclops*, and* Mesocyclops*) and marine (*Oithona* and* Paracyclopina*) organisms. Specific freshwater species were selected for analysis because these species are important for the maintenance of food chains in Russian freshwater ecosystems. The aim of this study was to analyze the sequence characteristics of the rDNA 28S gene, ITS1, and ITS2 regions as phylogenetic markers for the selected group of organisms.

## 2. Materials and Methods

### 2.1. Samples Collection

Nine freshwater species of the Cyclopidae family were collected near the Borok settlement in the Yaroslavskaya region of Russia:* Mesocyclops leuckarti* (Claus, 1857),* Cyclops strenuus* (Fischer, 1851), and* Cyclops insignis* (Claus, 1857) (population no. 1) from the Barskiy Pond (58°3′59.35′′N; 38°15′10.16′′E);* Thermocyclops oithonoides* (Sars, 1863) from the Sunoga pond (58°2′34.66′′N; 38°14′41.29′′E);* Thermocyclops crassus* (Fischer, 1853),* Macrocyclops distinctus* (Richard, 1887),* Macrocyclops albidus* (Jurine, 1820),* Diacyclops bicuspidatus* (Claus, 1857), and* Megacyclops viridis* (Jurine, 1820) (population no. 1) from the Ikhteologichesky Canal (58°3′55.62′′N; 38°15′21.05′′E); and* Megacyclops viridis* (Jurine, 1820) (population no. 2) from a pond in the flood zone of the Rybinsk Reservoir (58°4′4.70′′N; 38°15′39.88′′E).

*Cyclops kolensis* (Lilljeborg, 1901) and* Cyclops insignis* (Claus, 1857) (population no. 2) were collected from the Andreevsky small pond in Vorob'evy Gory, Moscow, Russia (55°42′35.40′′N; 37°34′6.61′′E). Two marine species,* Oncaea* sp. (Claus) and* Oithona similis *(Claus, 1866), were collected from the Norwegian Sea (68°52′36.67′′N; 3°8′21.91′′E).

Individuals of each species were collected for further analysis at the specified locations over a 0.5- to 1-hour period.

No specific permission was required to collect samples at these locations. None of the studied species is endangered or protected.

A fragment of the 28S gene from each of the four marine species* Paracyclopina nana* (Smirnov, 1935) (GenBank accession number FJ214952),* Oithona nana* (Giesbrecht, 1893) (GenBank accession number FM991727),* Oithona simplex* (Farran, 1913) (GenBank accession number AF385458), and* Oithona helgolandica* (Claus, 1863) (GenBank accession number FM991724.1) was also used for the analysis. The DNA was extracted from either samples preserved in 70% ethanol or raw materials (Moscow populations).

### 2.2. DNA Extraction, PCR Amplification, and Sequencing

The genomic DNA was isolated from 10–20 individuals of each collected species using the DNeasy Blood & Tissue kit (QIAGEN, Hilden, Germany) according to the manufacturer's instructions and was frozen at −20°C. The rDNA region (approximately 2000 bp) was amplified from the genomic DNA by polymerase chain reaction (PCR) using the universal eukaryotic rDNA primers DAMS18 and DAMS28 [95–97] (Figure 1). The amplified rDNA regions contained the ITS1 (261–388 bp) and ITS2 (188–262 bp) regions, the 5.8S gene (157 bp), and approximately 200 and 1000 bp of the 18S and 28s genes, respectively. The amplification was performed in 50 *μ*L reactions using a PCR Master Mix (2X) (Fermentas, Vilnius, Lithuania) according to the manufacturer's instructions; the reactions were performed in a Primus 25 advanced Thermocycler (PEQLAB, Erlangen, Germany) using previously published rDNA-specific parameters [98]. The PCR products were resolved on 1.0% agarose gels, and DNA was extracted from the observed unique bands using the QIAquick Gel Extraction Kit (QIAGEN, Hilden, Germany). The extracted products were cloned into the pGEM-T Easy vector (Promega, USA), and the resulting plasmids were used to transform* Escherichia coli* JM109 competent cells (Promega, USA) according to the manufacturer's instructions. For each species, the amplified product and five clones were sequenced. Automated sequences were generated on an ABI PRISM 310 Genetic Analyzer according to Sanger et al. [99] with a BigDye Termination kit (Applied Biosystems, USA). The sequences generated in this study were deposited in GenBank under the accession numbers KF153689–KF153701.

### 2.3. Phylogenetic Analyses

The rDNA sequences were aligned using ClustalW 2.1 [100, 101] with some manual adjustments. The boundaries of the ITS1 and ITS2 regions and the 28S gene were identified by comparing the primer-delimited sequences against sequences in the GenBank database using BLAST analysis. The boundaries of the conserved sequences were considered to represent the 5.8S, 18S, and 28S gene flanking regions if they were 100% similar to the boundaries of rDNA sequences in the GenBank database. The initial sequence alignment flanked by DAMS18/DAMS28 primers was divided into ITS1 and ITS2 alignments. The rDNA genetic distances were estimated using the MEGA V5.2 software [102]. DAMBE (Data Analysis in Molecular Biology and Evolution) software was used to analyze substitution saturation [103–105]. This method computes the entropy-based index of substitution saturation and its critical value. If the index of substitution saturation (Iss) approaches 1 or if the Iss is not smaller than the critical Iss value (Iss.c), then sequences are considered to contain substantial saturation. As is known, the substitution saturation decreases phylogenetic information contained in sequences and has plagued the phylogenetic analysis involving deep branches. In the extreme case when sequences have experienced full substitution saturation, the similarity between the sequences will depend entirely on the similarity in nucleotide frequencies, which often does not reflect phylogenetic relationships [106].

The rDNA-based phylogenetic trees were estimated using probabilistic (maximum likelihood (ML), Bayesian) and distance (minimum evolution (ME)) methods [107–110]. ML and ME analyses of ITS1, ITS2, and 28S data were performed using the program MEGA V5.2. Branch support was assessed using the bootstrap method [111] (1,000 replicates) with the close-neighbor-interchange (CNI) algorithm at a search level of 1 for ME analysis and heuristic search for ML analysis. The Bayesian information criterion (BIC), as implemented in MEGA V5.2, was used to identify the best-fit model of sequence evolution for the trees estimated using ML. The evolutionary history was inferred using the ML method based on the general time reversible with the gamma distribution shape parameter (GTR+G) model for 28S and the Hasegawa-Kishino-Yano with gamma distribution shape parameter (HKY+G) model [112] for the ITS1, ITS2, and concatenated ITS1/ITS2 alignments. In addition to these methods, ITS1 and ITS2 alignments were constructed using the MAFFT version 7 (http://mafft.cbrc.jp/alignment/server/) [113] and Gblocks version 0.91b (http://www.phylogeny.fr/version2_cgi/one_task.cgi?task_type=gblocks) software programs [114–116] to eliminate poorly aligned and highly divergent regions. Default parameters were used for both of these methods. The Tamura 3-parameter model (T92) [117] and HKY with evolutionary invariable (HKY+I) for Gblocks-treated MAFFT ITS1 and ITS2 data, respectively, were used to infer evolutionary history inference using the ML method.

The Bayesian analysis was performed using MrBayes version 3.1.2 software [118, 119]. Two replicate analyses of 1 million generations each were performed for each dataset, with sampling every 10 generations. The hierarchical likelihood ratio test (hLRT) implemented in MrModeltest version 2.3 software [120] was used to identify the model of best fit (Hasegawa-Kishino-Yano with invariant sites and gamma distribution shape parameter (HKY+I+G) [112] for ITS1 and the HKY+G model for ITS2). Trees from the first 53,000 and 118,000 generations were discarded as burn-in for ITS1 and ITS2, respectively. The Bayesian tree was estimated from the majority-rule consensus of the post-burn-in trees.

A reticulogram [121] was constructed using the T-REX version 4.01a software [122] with the distance matrix computed using the Kimura 2-parameters model (ignoring missing bases); the weighted least-squares method was used for tree reconstruction [123], and addition of reticulation branches stopped when *K* = 1 branches were added.

Network reconstruction was performed using Splits Tree 4 version 4.11.3 software [65]. The neighbor-net network method and uncorrected* p*-distances were used to analyze and visualize reticulate relationships. All gaps were excluded for analysis. Network robustness was tested using 1,000 bootstrap replicates.

## 3. Results and Discussion

### 3.1. Characteristics and Analysis of an rDNA Sequence Dataset

In each species, the nucleotide sequences of the amplified rDNA region and five clones were not significantly different from each other. The frequency of variable nucleotides did not exceed the average rate of nucleotide substitutions caused by DNA polymerase errors, which is approximately one substitution per 1,000 nucleotides. The compared sequences contained both relatively evolutionarily conserved (fragments of 18S and 28S rDNA and the complete 5.8S rDNA) and evolutionary variable genomic regions (ITS1 and ITS2). For different taxa, the ITS1 and ITS2 sequences vary significantly among individuals at the inter- and intrapopulation levels; furthermore, these sequences can exhibit intragenomic variability [25, 41, 53, 54]. Recently, a high level of intrapopulation polymorphism of the 28S rDNA sequences was observed within* Oithona* spp. [22]. However, there are instances of strong evolutionary conservation of the 28S and ITS sequences [15, 23, 30, 34]. Notably, the* M. leuckarti* ITS2 sequence obtained in this study did not exhibit any nucleotide substitutions compared to the* M. leuckarti* ITS2 sequence described previously (GenBank accession number GQ848499) [42]. Therefore, the strong evolutionary conservation of ITS1 and ITS2 sequences is a characteristic feature of the copepod species analyzed in this study.

In this study, the applicability of different segments of rDNA containing the ITS1 and ITS2 regions, the 5.8S RNA gene, and fragments of the 18S and 28S rRNA genes was examined for reconstruction of the phylogenetic relationships among freshwater cyclopoid copepods. The 5.8S gene and the analyzed fragment of the 18S gene were not considered for phylogenetic reconstruction due to their short length and strong evolutionary conservation: only a few nucleotide substitutions were detected by comparing these sequences with evolutionary distant species (data not shown).

For 15 specimens of Cyclopoida species (including the two marine species), the average length of the 28S gene fragment sequenced was 1051 bp. We trimmed these sequences to 703 bp and compared them with the 28S gene sequences of marine species available in GenBank. These 703 bp of 28S rDNA sequences were aligned, and 342 variable sites were observed.* Oncaea* sp. (Oncaeidae family) was used as the out group.

The ITS1 sequence lengths varied from 267 to 388 bp among the 13 Cyclopidae specimens. The ITS1 sequence alignments possessed 442 characters, and among them, 283 were variable. The ITS2 sequence lengths varied from 188 to 262 bp among the 13 Cyclopidae specimens. ITS2 sequence alignment possessed 302 characters, and 190 were variable.

All alignment sets were examined for homogeneity of base frequencies and substitution saturation. The average base frequencies of the 28S gene fragment (*A* = 20.52, *C* = 25.33, *G* = 32.75, and *T* = 21.40%) differed from the ITS1 (*A* = 14.27, *C* = 30.68, *G* = 27.55, and *T* = 27.50%) and ITS2 (*A* = 13.08, *C* = 29.64, *G* = 30.08, and *T* = 27.19%) regions. Gaps were excluded while estimating the average base frequencies of the ITS sequences. Using the chi-squared test, no significant differences were observed in the base compositions of the 28S (*χ* ^2^ = 39.77, *df* = 54, and *P* = 0.93), ITS1 (*χ* ^2^ = 22.04, *df* = 36, and *P* = 0.97), and ITS2 (*χ* ^2^ = 22.65, *df* = 36, and *P* = 0.96) sequences among different taxa.

To analyze whether the divergence of 28S, ITS1, and ITS2 rDNA fragments among species was saturated, we performed a substitution saturation test and generated saturation plots. Using DAMBE, the substitution saturation test revealed an Iss value that was significantly (*P* < 0.001) lower than the Iss.c in all cases (Table 1). This result indicated the suitability of the data for phylogenetic analysis. The total numbers of transition and transversion substitutions were plotted individually against model-corrected maximum-likelihood pairwise distances for the 28S, ITS1, and ITS2 sequences (see Supplementary Figure 1 in Supplementary Material available online at http://dx.doi.org/10.1155/2014/926342). Using linear regression analysis on the 28S, ITS1, and ITS2 saturation graphs, the coefficients of determination (*R* ^2^) were calculated for both classes of substitutions: for transitions, the *R* ^2^ values were 0.79, 0.68, and 0.74 for the 28S, ITS1, and ITS2 sequences, respectively; for transversions, the *R* ^2^ values were 0.95, 0.93, and 0.91 for the 28S, ITS1, and ITS2 sequences, respectively. The *R* ^2^ values indicated that no less than 70% of the total variation in pairwise transitions and transversions could be explained by the linear relationship between pairwise distances and the total number of transitions and transversions. All saturation plots showed significant linear correlations (Supplementary Figure 1). Therefore, both transitions and transversions steadily accumulated as the corrected pairwise divergence increased, indicating that saturation was not reached.

### 3.2. Distance Analyses and Phylogenetic Tree Reconstruction

Phylogenetic analysis of the cyclopoid copepods species based on rDNA showed that the 28S rDNA sequences are informative for the phylogeny of both higher-level and closely related Copepoda species, whereas the ITS1 and ITS2 sequences are highly informative for reconstruction of the evolutionary history of closely related species. The ITS1 and ITS2 sequences are known to evolve more rapidly than the ribosomal RNA genes. Consistent with this observation, in this study, the pairwise ITS1/ITS2* p*-distances were significantly higher thanthe 28S* p-*distances (compare Tables 2 and 3). These data are consistent with other studies showing considerable variation in ITS1 and ITS2 divergence levels among different groups of copepods [40, 42, 52, 124, 125]. In this study, fragments of 28S rDNA sequences were used for the analysis of marine and freshwater cyclopoid copepods species, whereas ITS1 and ITS2 sequences were used exclusively for the analysis of freshwater cyclopoid copepods species.

**Figure 2:**
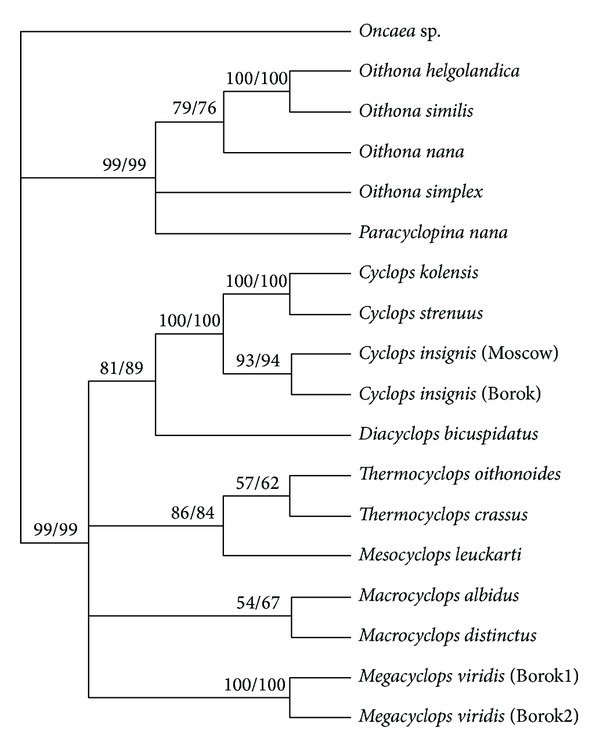
Phylogenetic relationships of Cyclopoida based on ~700 bp of the 28S rRNA gene. The consensus cladogram inferred from the 28S ribosomal DNA fragment sequence data of 16 Podoplea superorder species using maximum likelihood (ML) analysis under the HKY+G model and minimum evolution (ME) analysis. The numbers above branches indicate bootstrap percentages. The values are listed for ML/ME.

The cladogram based on comparison of the 28S rDNA sequences reflected the evolutionary history of the analyzed species (Figure 2).* Oncaea* sp. was used as the out group. Similar topologies and levels of support at most nodes were obtained for all 28S phylogenetic trees constructed using the ML and ME methods. The specimens belonging to the order Cyclopoida with high bootstrap support (ML/ME 99) formed two major clades on the tree (Figure 2). One clade combined the marine cyclopoid copepods species, whereas the freshwater species specimens formed the second clade. The* p*-distance between these two clades varied in the range of 0.171–0.245 (Table 2). The 28S phylogenetic tree revealed detailed relationships among the* Oithona* spp. with high bootstrap support (ML 79, 100 and ME 76, 100). However,* P. nana* (Cyclopettidae family) and* Oithona* spp. (Oithonidae family) were poorly resolved. Notably, this study is the second on the molecular phylogenetics of the* Oithona* spp.; the previous study described the phylogenetic relationships between three* Oithona* spp.:* O. similis*,* O. atlantica,* and* O. nana* [22].

**Figure 3:**
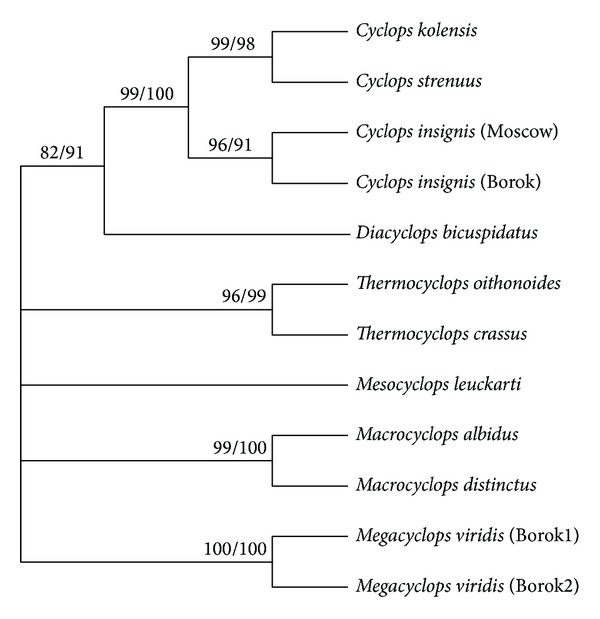
Phylogenetic relationships of Cyclopoida based on ~500 bp of concatenated ITS1/ITS2 rDNA sequences. The consensus cladogram inferred from the ITS1-ITS2 ribosomal DNA fragment sequence data of 10 species of the Cyclopidae family using maximum likelihood analysis under the HKY+G model and minimum evolution (ME) analysis. The numbers above branches indicate bootstrap percentages. The values are listed for ML/ME.

The cladogram based on the comparison of the concatenated ITS1/ITS2 sequences is shown in Figure 3. Notably, the 28S and ITS1/ITS2 cladograms had several common features, reflecting the evolutionary history of the analyzed freshwater cyclopoid copepods species. Both cladograms revealed that* D. bicuspidatus* and specimens of the* Cyclops* genus with high bootstrap values (>80) are separated from other studied freshwater copepods in a distinct clade. The* p*-distance between* D. bicuspidatus* and* Cyclops* spp. calculated based on ITS1/ITS2 analysis varied in the range of 0.232–0.250, whereas the* p*-distance between* D. bicuspidatus* and* Thermocyclops* spp. varied in the range of 0.298–0.333, and the* p*-distance between* D. bicuspidatus* and other analyzed freshwater species varied in the range of 0.310–0.405. This result is consistent with a previous phylogenetic study based on 18S rDNA sequence analysis [48]. Notably, the systematic position of this species, based solely on the analysis of morphological characteristics, remained unclear.* Diacyclops bicuspidatus* is considered to be evolutionarily closer to* Thermocyclops* spp. [8].

Another important conclusion from the analysis of 28S and ITS1/ITS2 cladograms relates to the systematic position of* C. strenuus*. The* Cyclops* genera subclade was divided into the* C. kolensis*:* C. strenuus* subsubclade and the* C. insignis* subsubclade (Figures 2 and 3). The* p*-distance between* C. strenuus* and* C. kolensis* calculated based on ITS1/ITS2 analysis is 0.006, whereas the* p*-distance between* C. strenuus* and* C. insignis *varied in the range of 0.042–0.054. Therefore,* C. strenuus* is more closely related to* C. kolensis* than to* C. insignis*. Notably, the phylogenetic relationships between the studied* Cyclops* species could not be elucidated solely on the basis of morphological characteristics.

The only difference between the 28S and ITS1/ITS2 cladograms within freshwater copepods was the position of* M. leuckarti* (Figures 2 and 3). The cladogram based on comparison of the 28S rDNA sequences showed that the* M. leuckarti* and* Thermocyclops* cluster together to form a separate subclade (Figure 2). This result is consistent with the previous observation that the* Mesocyclops* and* Thermocyclops* genera are phylogenetically closely related, which was confirmed by the similarity of morphological characteristics and using molecular data [42]. ITS1/ITS2 analysis revealed that* M. leuckarti* is located separately from* Thermocyclops* and other clades (Figure 3). Using phylogenetic networks, we analyzed whether the* M. leuckarti* position in the ITS1/ITS2 cladogram was caused by different contributions of ITS1 and ITS2 sequences to the phylogenetic signal.

### 3.3. Phylogenetic Networks

**Figure 4:**
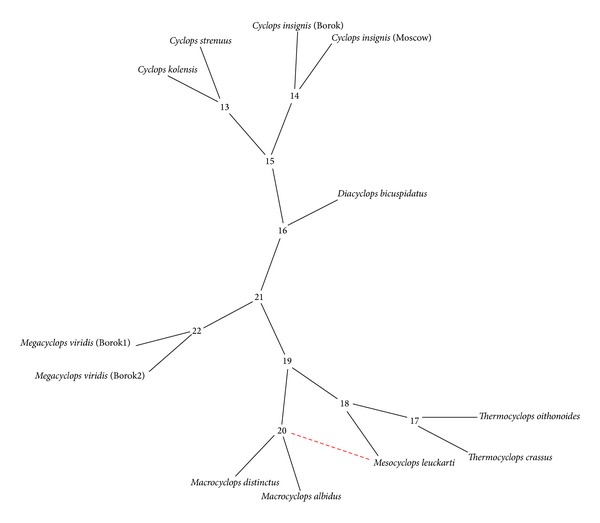
Reticulogram for concatenated ITS1/ITS2 sequences of 10 species of the Cyclopidae family. The red dashed line indicates the reticulation event connecting* M. leuckarti* to the* Macrocyclops* clade node. The number of internal vertices begins with *n* + 1, where *n* is the number of leaves. The order of internal vertices distribution corresponds to the increasing lengths of the 22 reticulogram edges.

A reticulogram-based phylogenetic network inference approach was used to verify the reticulate evolution of the studied copepods. Concatenated ITS1/ITS2 sequences of 10 species from the Cyclopidae family were used for reticulogram reconstruction. The reticulogram revealed a network with* Mesocyclops* and* Thermocyclops* clustered together and a reticulation (lateral branch) connecting* M. leuckarti* to the* Macrocyclops* clade node (Figure 4). Therefore, the reticulogram indicated the reticulation in* Mesocyclops* evolution.

**Figure 5:**
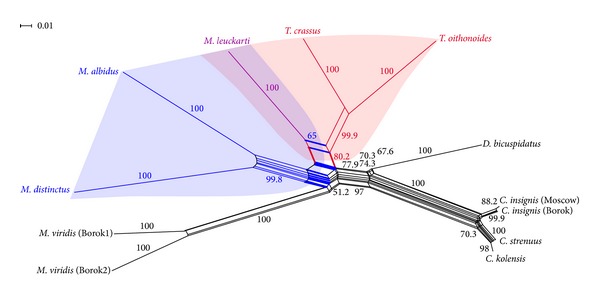
Split networks for concatenated ITS1/ITS2 sequences of 10 species of the Cyclopidae family. Split network based on concatenated ITS1/ITS2 sequences; the split separating* M. leuckarti*,* T. oithonoides,* and* T. crassus* is indicated in bold red; the split separating* M. leuckarti*,* M. distinctus,* and* M. albidus* is indicated in bold blue; purple indicates the* M. leuckarti *reticulate relationship with* Thermocyclops* and* Macrocyclops*. The values on the branches indicate bootstrap percentages.

A split network represents incompatible edges of trees as a band of parallel edges. Parallel edges split a network into two sets of nodes. Split-graph analysis of concatenated ITS1/ITS2 sequences of 10 species from the Cyclopidae family revealed a reticulate relationship between* Mesocyclops*,* Thermocyclops,* and* Macrocyclops* with high reliability (Figure 5). All principal splits were well supported. Two splits were observed in the ITS1/ITS2 split network. The first split (parallel edges highlighted with bold red) separated* M. leuckarti*,* T. oithonoides,* and* T. crassus* with 80.2% bootstrap support. The second split (parallel edges highlighted with bold blue) separated* M. leuckarti*,* M. distinctus*, and* M. albidus* with 65.0% bootstrap support.

**Figure 6:**
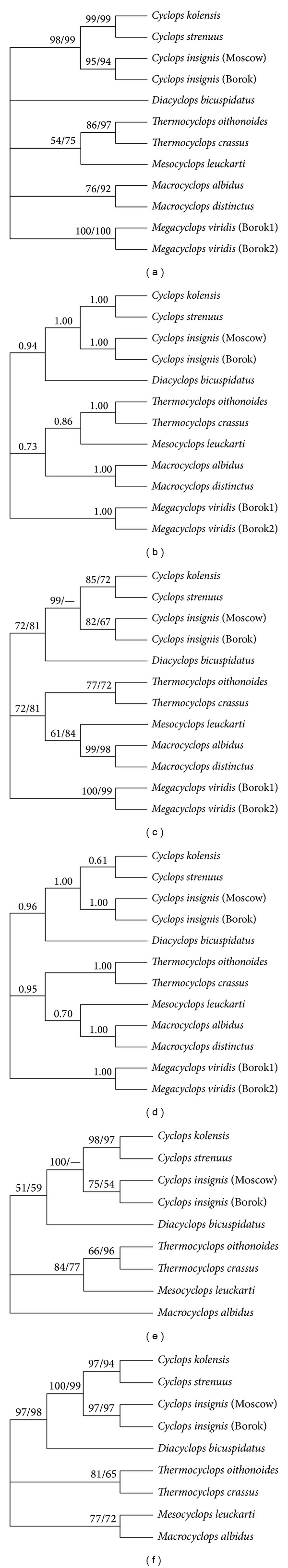
Phylogenetic relationships of Cyclopidae based on ITS1 and ITS2 sequences. The ITS1 consensus Clustal tree of ten Cyclopidae species constructed using (a) maximum likelihood and minimum evolution and (b) Bayesian inference. The ITS2 consensus Clustal tree of ten Cyclopidae species constructed using (c) maximum likelihood and minimum evolution and (d) Bayesian inference. The consensus Gblocks-treated MAFFT trees of eight Cyclopidae species constructed using maximum likelihood and minimum evolution: (e) ITS1, (f) ITS2. The numbers above the branches indicate bootstrap percentages and Bayesian posterior probabilities. The values are listed for ML/ME. Missing or weakly supported nodes (<50% or 0.5) are denoted by a ‘‘—”.

In addition to the network data, we performed phylogenetic reconstruction based on independent ITS1 and ITS2 analyses using probabilistic and distance methods. Irrespective of the method used, the main difference between the topologies of the ITS1 and ITS2 phylogenetic trees was as follows: based on the ITS1 analysis,* M. leuckarti* is clustered with* Thermocyclops*, whereas the ITS2 analysis revealed that* M. leuckarti* clustered with* Macrocyclops* (Figures 6(a)–6(d)).

The impact of the chosen DNA sequence on the clustering of* M. leuckarti* might reflect the different evolutionary histories of ITS1 and ITS2, which indicates the potential hybrid origin of* M. leuckarti*. However, the values of bootstrap support for the clustering of* Mesocyclops* and* Thermocyclops* and of* Mesocyclops* and* Macrocyclops* depended on the method used for phylogenetic tree reconstruction and varied over a wide range (Figures 6(a)–6(d)).

Phylogenetic trees can be inconsistent due to the so-called long-branch attraction (LBA) phenomenon, which occurs when two nonadjacent taxa share many homoplastic character states along long branches and/or from uncorrected sequence alignments. Interpretation of the observed similarity depends on the method used for phylogenetic analysis, and this similarity can often be interpreted as homology. Model-based methods are most resistant to LBA, but these methods can exhibit LBA if their assumptions are seriously violated or if there are insufficient taxa in the analysis to accurately estimate the parameters of the evolutionary model [126]. Taxon sampling is a crucial factor for avoiding LBA in phylogenetic analysis [127]. The inclusion of additional taxa in phylogenetic analysis increases the accuracy of the inferred topology by dispersing homoplasty across the tree and reducing the effect of LBA. The LBA effect might also be revealed by exclusion of the long-branched taxon from the analysis [127].

To reduce the possible effects of LBA and correctness of the sequence alignment, we used the following approaches: (1) three taxa the most evolutionarily distant from* M. leuckarti* (*M. distinctus*,* M. viridis* Borok1, and* M. viridis* Borok2) were removed from the list of species used for ITS1 and ITS2 phylogenetic tree reconstruction (the taxa selection was based on the data presented in Table 3) and (2) ITS1 and ITS2 sequences were aligned using new multiple sequence alignment programs to eliminate poorly aligned and highly divergent regions (see Section 2). The final ITS1 and ITS2 alignments are shown in Supplementary Figures 2(a) and 2(b), and the resulting phylogenetic trees are shown in Figures 6(e) and 6(f). Based on the ITS1 analysis,* M. leuckarti* clustered with* Thermocyclops*, whereas the ITS2 analysis revealed that* M. leuckarti* clustered with* Macrocyclops* (Figures 6(e) and 6(f)). Notably, the topology of the new phylogenetic trees had high bootstrap support: 84 (ML)/77 (ME) for ITS1 and 77 (ML)/72 (ME) for ITS2 (Figures 6(e) and 6(f)).

We think that one of the most intriguing explanations for the observed differences in the clustering of* M. leuckarti* is the interspecific hybridization between extinct taxa (presumably closely related) that were ancestral to both* Mesocyclops*,* Macrocyclops*, and* Thermocyclops*. However, a rigorous proof of this hypothesis requires further analysis of a larger number of species. This will be the subject of our further research.

## 4. Conclusion

We evaluated the utility of a ~2000 bp fragment of rDNA (easily amplified by universal primers) for the phylogenetic reconstruction of the relationships of Copepoda species. Our data showed that the 28S rDNA and the ITS1 and ITS2 regions are highly informative for the phylogeny of both higher-level and closely related Copepoda species. Comparative analysis of the ITS1 and ITS2 nucleotide sequences among closely related Copepoda species revealed an unusual evolutionary history of these spacer sequences; therefore, the ITS1 and ITS2 regions might contain different phylogenetic signals.

## Supplementary Material

The Supplementary Material contains figures of substitution saturation plots of 28S, ITS1, ITS2, which allow to estimate the quality of phylogenetic signals; and ITS1 and ITS2 alignments of 8 species built in Mafft v.7 and treated by Gblocks v.0.91b, which give notion about sequences variability.

## Figures and Tables

**Table 1:** False test of substitution saturation.

Alignment	Iss	Iss.c	Std. error
28S	0.200	0.739	0.019
ITS1	0.427	0.679	0.044
ITS2	0.376	0.665	0.043

Testing whether the observed Iss is significantly (*P* < 0.001, two-tailed *t*-test) lower than the Iss.c for a symmetrical tree.

**Table 2:** Pairwise uncorrected genetic distance among 18 sequences of Cyclopoida based on comparison of 28S rRNA gene.

#	Species name	1	2	3	4	5	6	7	8	9	10	11	12	13	14	15	16	17
1	*Oncaea *sp.																	
2	*Paracyclopina nana *	0.229																
3	*Oithona similis *	0.230	0.148															
4	*Oithona helgolandica *	0.227	0.143	0.011														
5	*Oithona nana *	0.233	0.181	0.155	0.146													
6	*Oithona simplex *	0.244	0.152	0.175	0.166	0.177												
7	*Cyclops kolensis *	0.209	0.223	0.197	0.195	0.244	0.212											
8	*Cyclops strenuus *	0.207	0.220	0.197	0.195	0.242	0.207	0.005										
9	*Cyclops insignis *Moscow	0.216	0.223	0.204	0.203	0.245	0.201	0.037	0.032									
10	*C. insignis* Borok	0.209	0.216	0.198	0.197	0.241	0.197	0.027	0.023	0.014								
11	*Diacyclops bicuspidatus *	0.186	0.200	0.183	0.180	0.224	0.194	0.090	0.085	0.098	0.087							
12	*Thermocyclops oithonoides *	0.220	0.203	0.191	0.186	0.216	0.184	0.128	0.123	0.120	0.116	0.098						
13	*Thermocyclops crassus *	0.218	0.203	0.181	0.178	0.220	0.204	0.127	0.125	0.127	0.125	0.093	0.084					
14	*Mesocyclops leuckarti *	0.198	0.183	0.174	0.171	0.215	0.181	0.133	0.128	0.123	0.122	0.090	0.087	0.079				
15	*Macrocyclops albidus *	0.221	0.198	0.197	0.191	0.215	0.178	0.145	0.143	0.137	0.134	0.099	0.131	0.114	0.105			
16	*Macrocyclops distinctus *	0.230	0.216	0.192	0.192	0.218	0.191	0.152	0.148	0.143	0.142	0.113	0.127	0.143	0.131	0.123		
17	*Megacyclops viridis* Borok1	0.213	0.207	0.195	0.192	0.230	0.191	0.137	0.134	0.134	0.123	0.105	0.116	0.117	0.114	0.130	0.139	
18	*M. viridis* Borok2	0.207	0.210	0.206	0.203	0.236	0.198	0.123	0.122	0.127	0.119	0.085	0.114	0.110	0.105	0.125	0.130	0.050

All positions containing gaps and missing data were eliminated (complete deletion option). Final dataset, 657 positions.

**Table 3:** Pairwise uncorrected genetic distance among 12 Cyclopidae sequences based on comparison of concatenated ITS1/ITS2 rRNA sequences.

#	Species name	1	2	3	4	5	6	7	8	9	10	11
1	*Cyclops kolensis *											
2	*Cyclops strenuus *	0.006										
3	*Cyclops insignis* Moscow	0.054	0.048									
4	*C. insignis* Borok	0.048	0.042	0.006								
5	*Diacyclops bicuspidatus *	0.250	0.244	0.232	0.232							
6	*Thermocyclops oithonoides *	0.333	0.330	0.330	0.330	0.313						
7	*Thermocyclops crassus *	0.301	0.298	0.295	0.295	0.262	0.188					
8	***Mesocyclops leuckarti***	0.324	0.321	0.310	0.310	0.286	0.280	0.241				
9	*Macrocyclops distinctus* ∗	0.414	0.411	0.408	0.405	0.393	0.429	0.408	0.369∗			
10	*Macrocyclops albidus *	0.393	0.390	0.381	0.378	0.357	0.369	0.366	0.336	0.321		
11	*Megacyclops viridis* Borok1∗	0.381	0.384	0.396	0.390	0.387	0.387	0.387	0.390∗	0.455	0.438	
12	*M. viridis* Borok2∗	0.375	0.378	0.384	0.378	0.363	0.366	0.357	0.381∗	0.438	0.429	0.149

All positions containing gaps and missing data were eliminated (complete deletion option). Final datasets, 336 positions for concatenated ITS1/ITS2. The names of the three taxa most evolutionarily distant from* Mesocyclops* and the values of the corresponding genetic distances are shown with asterisk.

## References

[1] Boxshall GA, Defaye D (2008). Global diversity of copepods (Crustacea: Copepoda) in freshwater. *Hydrobiologia*.

[2] Dussart B, Defaye D (2006). *World Directory of Crustacea Copepoda of Inland Waters. II—Cyclopiformes*.

[3] Kiefer F (1927). Versuch eines Systems der Cyclopiden. *Zoologischer Anzeiger*.

[4] Gurney R (1933). *British Fresh-Water Copepoda. III. Cyclopoida*.

[5] Rylov VM (1948). *Cyclopoida presnykh vod. Freshwater Cyclopoida V.3*.

[6] Yeatman HC, Edmondson WT (1959). Free-living Copepoda. Cyclopoida. *Fresh-water Biology*.

[7] Dussart B (1969). *Les Copépodes des Eaux Continentales de Europe Occidentals, Vol. 2. Cyclopoides et Biologie*.

[8] Monchenko VI (1974). Gnathostomata cyclopoida: cyclopidae. *The Fauna of Ukraina Naukova Dumka, Kiev, USSR*.

[9] Bucklin A, Frost BW, Kocher TD (1992). DNA sequence variation of the mitochondrial 16S rRNA in *Calanus* (Copepoda: Calanoida): intraspecific and interspecific patterns. *Molecular Marine Biology and Biotechnology*.

[10] Bucklin A, LaJeunesse TC, Curry E, Wallinga J, Garrison K (1996). Molecular diversity of the copepod, *Nannocalanus minor*: genetic evidence of species and population structure in the North Atlantic Ocean. *Journal of Marine Research*.

[11] Burton RS (1998). Intraspecific phylogeography across the point conception biogeographic boundary. *Evolution*.

[12] Caudill CC, Bucklin A (2004). Molecular phylogeography and evolutionary history of the estuarine copepod, *Acartia tonsa*, on the Northwest Atlantic coast. *Hydrobiologia*.

[13] Edmands S (2001). Phylogeography of the intertidal copepod *Tigriopus californicus* reveals substantially reduced population differentiation at northern latitudes. *Molecular Ecology*.

[14] Eyun S, Lee Y, Suh H, Kim S, Ho YS (2007). Genetic identification and molecular phylogeny of *Pseudodiaptomus* species (Calanoida, Pseudodiaptomidae) in Korean waters. *Zoological Science*.

[15] Goetze E (2011). Population differentiation in the open sea: insights from the pelagic copepod pleuromamma xiphias. *Integrative and Comparative Biology*.

[16] Laakmann S, Holst S (2014). Emphasizing the diversity of North Sea hydromedusae by combined morphological and molecular methods. *Journal of Plankton Research*.

[17] Lindeque PK, Harris RP, Jones MB, Smerdon GR (2007). Distribution of *Calanus* spp. as determined using a genetic identification system. *Scientia Marina*.

[18] Minxiao W, Song S, Chaolun L, Xin S (2011). Distinctive mitochondrial genome of Calanoid copepod *Calanus sinicus* with multiple large non-coding regions and reshuffled gene order: useful molecular markers for phylogenetic and population studies. *BMC Genomics*.

[19] Rawson PD, Burton RS (2006). Molecular evolution at the cytochrome oxidase subunit 2 gene among divergent populations of the intertidal copepod, *Tigriopus californicus*. *Journal of Molecular Evolution*.

[20] Soh HY, Ok Park E, Venmathi Maran BA, Yong Moon S (2013). A new species of Acartia subgenus Euacartia (Copepoda: Calanoida: Acartiidae) from Korean estuaries based on morphological and molecular evidence. *Journal of Crustacean Biology*.

[21] Willett CS, Ladner JT (2009). Investigations of fine-scale phylogeography in *Tigriopus californicus* reveal historical patterns of population divergence. *BMC Evolutionary Biology*.

[22] Cepeda GD, Blanco-Bercial L, Bucklin A, Berón CM, Viñas MD (2012). Molecular systematic of three species of Oithona (Copepoda, Cyclopoida) from the Atlantic ocean: comparative analysis using 28S rDNA. *PLoS ONE*.

[23] Hirai J, Shimode S, Tsuda A (2013). Evaluation of ITS2-28S as a molecular marker for identification of calanoid copepods in the subtropical western North Pacific. *Journal of Plankton Research*.

[24] Kim J, Kim W (2000). Molecular phylogeny of poecilostome copepods based on the 18S rDNA sequences. *Korean Journal of Biological Sciences*.

[25] Shinn AP, Banks BA, Tange N (2000). Utility of 18S rDNA and ITS sequences as population markers for *Lepeophtheirus salmonis* (Copepoda: Caligidae) parasitising *Atlantic salmon* (Salmo salar) in Scotland. *Contributions to Zoology*.

[26] Adamowicz SJ, Menu-Marque S, Halse SA (2010). The evolutionary diversification of the Centropagidae (Crustacea, Calanoida): a history of habitat shifts. *Molecular Phylogenetics and Evolution*.

[27] Blanco-Bercial L, Bradford-Grieve J, Bucklin A (2011). Molecular phylogeny of the Calanoida (Crustacea: Copepoda). *Molecular Phylogenetics and Evolution*.

[28] Braga E, Zardoya R, Meyer A, Yen J (1999). Mitochondrial and nuclear rRNA based copepod phylogeny with emphasis on the Euchaetidae (Calanoida). *Marine Biology*.

[29] Bucklin A, Frost BW, Bradford-Grieve J, Allen LD, Copley NJ (2003). Molecular systematic and phylogenetic assessment of 34 calanoid copepod species of the Calanidae and Clausocalanidae. *Marine Biology*.

[30] Bucklin A, Frost BW (2009). Morphological and molecular Phylogenetic analysis of evolutionary lineages within *Clausocalanus* (Copepoda: Calanoida). *Journal of Crustacean Biology*.

[31] Cornils A, Blanco-Bercial L (2013). Phylogeny of the paracalanidae giesbrecht, 1888 (Crustacea: Copepoda: Calanoida). *Molecular Phylogenetics and Evolution*.

[32] Dippenaar SM (2009). Estimated molecular phylogenetic relationships of six siphonostomatoid families (Copepoda) symbiotic on elasmobranchs. *Crustaceana*.

[33] Figueroa DF (2011). Phylogenetic analysis of *Ridgewayia* (Copepoda: Calanoida) from the Galapagos and of a new species from the Florida keys with a reevaluation of the phylogeny of Calanoida. *Journal of Crustacean Biology*.

[34] Goetze E (2003). Cryptic speciation on the high seas; global phylogenetics of the copepod family Eucalanidae. *Proceedings of the Royal Society B: Biological Sciences*.

[35] Laakmann S, Gerdts G, Erler R, Knebelsberger T, Martínez Arbizu P, Raupach MJ (2013). Comparison of molecular species identification for North Sea calanoid copepods (Crustacea) using proteome fingerprints and DNA sequences. *Molecular Ecology Resources*.

[36] Machida RJ, Miya MU, Nishida M, Nishida S (2006). Molecular phylogeny and evolution of the pelagic copepod genus *Neocalanus* (Crustacea: Copepoda). *Marine Biology*.

[37] Taniguchi M (2004). Molecular phylogeny of *Neocalanus* copepods in the subarctic Pacific Ocean, with notes on non-geographical genetic variations for *Neocalanus cristatus*. *Journal of Plankton Research*.

[38] Lee CE (2000). Global phylogeography of a cryptic copepod species complex and reproductive isolation between genetically proximate ‘populations’. *Evolution*.

[39] Adamowicz SJ, Menu-Marque S, Hebert PDN, Purvis A (2007). Molecular systematics and patterns of morphological evolution in the Centropagidae (Copepoda: Calanoida) of Argentina. *Biological Journal of the Linnean Society*.

[40] Goetze E (2005). Global population genetic structure and biogeography of the oceanic copepods *Eucalanus hyalinus* and *E. spinifer*. *Evolution*.

[41] Machida RJ, Tsuda A (2010). Dissimilarity of species and forms of planktonic *Neocalanus* copepods using mitochondrial COI, 12S, nuclear ITS, and 28S gene sequences. *PLoS ONE*.

[42] Wyngaard GA, Hołyńska M, Schulte JA (2010). Phylogeny of the freshwater copepod *Mesocyclops* (Crustacea: Cyclopidae) based on combined molecular and morphological data, with notes on biogeography. *Molecular Phylogenetics and Evolution*.

[43] Alekseev V, Dumont HJ, Pensaert J, Baribwegure D, Vanfleteren JR (2006). A redescription of *Eucyclops serrulatus* (Fischer, 1851) (Crustacea: Copepoda: Cyclopoida) and some related taxa, with a phylogeny of the *E. serrulatus*-group. *Zoologica Scripta*.

[44] Miracle MR, Alekseev V, Monchenko V, Sentandreu V, Vicente E (2013). Molecular-genetic-based contribution to the taxonomy of the *Acanthocyclops robustus* group. *Journal of Natural History*.

[45] Bláha M, Hulák M, Slouková J, Těšitel J (2010). Molecular and morphological patterns across *Acanthocyclops vernalis-robustus* species complex (Copepoda, Cyclopoida). *Zoologica Scripta*.

[46] Karanovic T, Krajicek M (2012). When anthropogenic translocation meets cryptic speciation globalized bouillon originates; molecular variability of the cosmopolitan freshwater cyclopoid *Macrocyclops albidus* (Crustacea: Copepoda). *Annales de Limnologie*.

[47] Karanovic T, Krajicek M (2012). First molecular data on the Western Australian *Diacyclops* (Copepoda, Cyclopoida) confirm morpho-species but question size differentiation and monophyly of the *Alticola*-group. *Crustaceana*.

[48] Mayor TY, Sheveleva NG, Sukhanova LV, Timoshkin OA, Kiril'chik SV (2010). Molecular-phylogenetic analysis of cyclopoids (Copepoda: Cyclopoida) from Lake Baikal and its water catchment basin. *Russian Journal of Genetics*.

[49] Karanovic T, Cooper SJB (2011). Molecular and morphological evidence for short range endemism in the *Kinnecaris solitaria* complex (Copepoda: Parastenocarididae), with descriptions of seven new species. *Zootaxa*.

[50] Marrone F, Brutto SL, Arculeo M (2010). Molecular evidence for the presence of cryptic evolutionary lineages in the freshwater copepod genus *Hemidiaptomus* G.O. Sars, 1903 (Calanoida, Diaptomidae). *Hydrobiologia*.

[51] Scheihing R, Cardenas L, Nespolo RF (2010). Morphological and molecular analysis of centropagids from the high Andean plateau (Copepoda: Calanoidea). *Hydrobiologia*.

[52] Thum RA, Harrison RG (2009). Deep genetic divergences among morphologically similar and parapatric *Skistodiaptomus* (Copepoda: Calanoida: Diaptomidae) challenge the hypothesis of Pleistocene speciation. *Biological Journal of the Linnean Society*.

[53] Soh HY, Kwon SW, Lee W, Yoon YH (2012). A new *Pseudodiaptomus* (Copepoda, Calanoida) from Korea supported by molecular data. *Zootaxa*.

[54] Marszalek MA, Dayanandan S, Maly EJ (2009). Phylogeny of the genus *Hesperodiaptomus* (Copepoda) based on nucleotide sequence data of the nuclear ribosomal gene. *Hydrobiologia*.

[55] Thum RA (2004). Using 18S rDNA to resolve diaptomid copepod (Copepoda: Calanoida: Diaptomidae) phylogeny: an example with the North American genera. *Hydrobiologia*.

[56] Huys R, Llewellyn-Hughes J, Olson PD, Nagasawa K (2006). Small subunit rDNA and Bayesian inference reveal *Pectenophilus ornatus* (Copepoda *incertae sedis*) as highly transformed Mytilicolidae, and support assignment of Chondracanthidae and Xarifiidae to Lichomolgoidea (Cyclopoida). *Biological Journal of the Linnean Society*.

[57] Huys R, Llewellyn-Hughes J, Conroy-Dalton S, Olson PD, Spinks JN, Johnston DA (2007). Extraordinary host switching in siphonostomatoid copepods and the demise of the Monstrilloida: integrating molecular data, ontogeny and antennulary morphology. *Molecular Phylogenetics and Evolution*.

[58] Ki J, Lee K, Park HG, Chullasorn S, Dahms H, Lee J (2009). Phylogeography of the copepod *Tigriopus japonicus* along the Northwest Pacific rim. *Journal of Plankton Research*.

[59] Hebert PDN, Cywinska A, Ball SL, DeWaard JR (2003). Biological identifications through DNA barcodes. *Proceedings of the Royal Society B*.

[60] Machida RJ, Miya MU, Nishida M, Nishida S (2002). Complete mitochondrial DNA sequence of *Tigriopus japonicus* (Crustacea: Copepoda). *Marine Biotechnology*.

[61] Burton RS, Byrne RJ, Rawson PD (2007). Three divergent mitochondrial genomes from California populations of the copepod *Tigriopus californicus*. *Gene*.

[62] Gerbi SA (1985). Evolution of ribosomal DNA. *Molecular Evolutionary Genetics*.

[63] Hwang UW, Kim W (1999). General properties and phylogenetic utilities of nuclear ribosomal DNA and mitochondrial DNA commonly used in molecular systematics.. *Korean Journal of Parasitology*.

[64] Hillis DM, Dixon MT (1991). Ribosomal DNA: molecular evolution and phylogenetic inference. *Quarterly Review of Biology*.

[65] Huson DH, Bryant D (2006). Application of phylogenetic networks in evolutionary studies. *Molecular Biology and Evolution*.

[66] Hillis DM, Moritz C, Mable BK (1996). *Molecular Systematics*.

[67] Koonin EV (2012). *The Logic of Chance: The Nature and Origin of Biological Evolution*.

[68] Sang T, Crawford DJ, Stuessy TF (1995). Documentation of reticulate evolution in peonies (*Paeonia*) using internal transcribed spacer sequences of nuclear ribosomal DNA: implications for biogeography and concerted evolution. *Proceedings of the National Academy of Sciences of the United States of America*.

[69] Giessler S, Englbrecht CC (2009). Dynamic reticulate evolution in a *Daphnia* multispecies complex. *Journal of Experimental Zoology A, Ecological Genetics and Physiology*.

[70] Kauserud H, Schumacher T (2003). Ribosomal DNA variation, recombination and inheritance in the basidiomycete *Trichaptum abietinum*: implications for reticulate evolution. *Heredity*.

[71] Wyatt PMW, Pitts CS, Butlin RK (2006). A molecular approach to detect hybridization between bream *Abramis brama*, roach *Rutlius rutilus* and rudd *Scardinius erythrophthalmus*. *Journal of Fish Biology*.

[72] Fuertes Aguilar J, Nieto Feliner G (2003). Additive polymorphisms and reticulation in an ITS phylogeny of thrifts (*Armeria*, Plumbaginaceae). *Molecular Phylogenetics and Evolution*.

[73] Yamaji H, Fukuda T, Yokoyama J (2007). Reticulate evolution and phylogeography in *Asarum*sect. *Asiasarum* (Aristolochiaceae) documented in internal transcribed spacer sequences (ITS) of nuclear ribosomal DNA. *Molecular Phylogenetics and Evolution*.

[74] Hershkovitz MA, Hernández-Pellicer CC, Arroyo MTK (2006). Ribosomal DNA evidence for the diversification of *Tropaeolum* sect. *Chilensia* (Tropaeolaceae). *Plant Systematics and Evolution*.

[75] Hugall A, Stanton J, Moritz C (1999). Reticulate evolution and the origins of ribosomal internal transcribed spacer diversity in apomictic *Meloidogyne*. *Molecular Biology and Evolution*.

[76] Wendel JF, Schnabel A, Seelanan T (1995). Bidirectional interlocus concerted evolution following allopolyploid speciation in cotton (*Gossypium*). *Proceedings of the National Academy of Sciences of the United States of America*.

[77] O'Donnell K, Cigelnik E (1997). Two divergent intragenomic rDNA ITS2 types within a monophyletic lineage of the fungus *Fusarium* are nonorthologous. *Molecular Phylogenetics and Evolution*.

[78] O'Donnell K, Cigelnik E, Nirenberg HI (1998). Molecular systematics and phylogeography of the *Gibberella fujikuroi* species complex. *Mycologia*.

[79] Brasier CM, Cooke DEL, Duncan JM (1999). Origin of a new *Phytophthora* pathogen through interspecific hybridization. *Proceedings of the National Academy of Sciences of the United States of America*.

[80] Hughes KW, Petersen RH (2001). Apparent recombination or gene conversion in the ribosomal ITS region of a *Flammulina* (Fungi, Agaricales) hybrid. *Molecular Biology and Evolution*.

[81] Newcombe G, Stirling B, McDonald S, Bradshaw HD (2000). *Melampsora* × *columbiana*, a natural hybrid of *M. medusae* and *M. occidentalis*. *Mycological Research*.

[82] Chu KH, Li CP, Ho HY (2001). The first internal transcribed spacer (ITS-1) of ribosomal DNA as a molecular marker for phylogenetic and population analyses in crustacea. *Marine Biotechnology*.

[83] Rieseberg LH (1997). Hybrid origins of plant species. *Annual Review of Ecology and Systematics*.

[84] Cui R, Schumer M, Kruesi K, Walter R, Andolfatto P, Rosenthal GG (2013). Phylogenomics reveals extensive reticulate evolution in *Xiphophorus* fishes. *Evolution*.

[85] Bullini L (1994). Origin and evolution of animal hybrid species. *Trends in Ecology and Evolution*.

[86] Dowling TE, Secor CL (1997). The role of hybridization and introgression in the diversification of animals. *Annual Review of Ecology and Systematics*.

[87] Mallet J (2005). Hybridization as an invasion of the genome. *Trends in Ecology and Evolution*.

[88] Arnason U, Spilliaert R, Pálsdóttir A, Arnason A (1991). Molecular identification of hybrids between the two largest whale species, the blue whale (*Balaenoptera musculus*) and the fin whale (*B. physalus*). *Hereditas*.

[89] Brower AVZ (2013). Introgression of wing pattern alleles and speciation via homoploid hybridization in *Heliconius butterflies*: a review of evidence from the genome.. *Proceedings Biological Sciences*.

[90] Carson HL, Kaneshiro KY, Val FC (1989). Natural hybridization between the sympatric Hawaiian species *Drosophila silvestris* and *Drosophila heteroneura*. *Evolution*.

[91] Gießler S, Englbrecht CC (2009). Dynamic reticulate evolution in a *Daphnia* multispecies complex. *Journal of Experimental Zoology A: Ecological Genetics and Physiology*.

[92] Gießler S, Mader E, Schwenk K (1999). Morphological evolution and genetic differentiation in *Daphnia* species complexes. *Journal of Evolutionary Biology*.

[93] Taylor DJ, Hebert PDN, Colbourne JK (1996). Phylogenetics and evolution of the *Daphnia longispina* group (Crustacea) based on 12S rDNA sequence and allozyme variation. *Molecular Phylogenetics and Evolution*.

[94] Vergilino R, Markova S, Ventura M, Manca M, Dufresne F (2011). Reticulate evolution of the *Daphnia pulex* complex as revealed by nuclear markers. *Molecular Ecology*.

[95] Mukha DV, Sidorenko AP (1995). Detection and analysis of *Tetrahymena pyriformis* 26S ribosomal DNA domain sequences, differing in degree of evolutionary conservation. *Molekulyarnaya Biologiya*.

[96] Mukha DV, Sidorenko AP (1996). Identification of highly conservative domains within the 17s ribosomal dna sequence from *Tetrahymena pyriformis*. *Genetika*.

[97] Mukha DV, Sidorenko AP, Lazebnaya IV, Wiegmann BM, Schal C (2000). Analysis of intraspecies polymorphism in the ribosomal DNA cluster of the cockroach *Blattella germanica*. *Insect Molecular Biology*.

[98] Mukha D, Wiegmann BM, Schal C (2002). Evolution and phylogenetic information content of the ribosomal DNA repeat unit in the Blattodea (Insecta). *Insect Biochemistry and Molecular Biology*.

[99] Sanger F, Nicklen S, Coulson AR (1977). DNA sequencing with chain-terminating inhibitors. *Proceedings of the National Academy of Sciences of the United States of America*.

[100] Larkin MA, Blackshields G, Brown NP (2007). Clustal W and Clustal X version 2.0. *Bioinformatics*.

[101] Goujon M, McWilliam H, Li W (2010). A new bioinformatics analysis tools framework at EMBL-EBI. *Nucleic Acids Research*.

[102] Tamura K, Peterson D, Peterson N, Stecher G, Nei M, Kumar S (2011). MEGA5: molecular evolutionary genetics analysis using maximum likelihood, evolutionary distance, and maximum parsimony methods. *Molecular Biology and Evolution*.

[103] Xia X, Xie Z (2001). DAMBE: software package for data analysis in molecular biology and evolution. *Journal of Heredity*.

[104] Xia X (2000). *Data Analysis in Molecular Biology and Evolution*.

[105] Xia X, Xie Z, Salemi M, Chen L, Wang Y (2003). An index of substitution saturation and its application. *Molecular Phylogenetics and Evolution*.

[106] Lemey P, Salemi M, Vandamme A-M (2009). *The Phylogenetic Handbook: A Practical Approach to Phylogenetic Analysis and Hypothesis Testing*.

[107] Fitch WM (1971). Toward defining the course of evolution: minimum change for a specific tree topology. *Systematic Biology*.

[108] Felsenstein J (1981). Evolutionary trees from DNA sequences: a maximum likelihood approach. *Journal of Molecular Evolution*.

[109] Rzhetsky A, Nei M (1992). A simple method for estimating and testing minimum-evolution trees. *Molecular Biology and Evolution*.

[110] Huelsenbeck JP, Ronquist F, Nielsen R, Bollback JP (2001). Bayesian inference of phylogeny and its impact on evolutionary biology. *Science*.

[111] Felsenstein J (1985). Confidence limits on phylogenies: an approach using the bootstrap. *Evolution*.

[112] Hasegawa M, Kishino H, Yano T (1985). Dating of the human-ape splitting by a molecular clock of mitochondrial DNA. *Journal of Molecular Evolution*.

[113] Katoh K, Kuma K, Toh H, Miyata T (2005). MAFFT version 5: improvement in accuracy of multiple sequence alignment. *Nucleic Acids Research*.

[114] Dereeper A, Audic S, Claverie J, Blanc G (2010). BLAST-EXPLORER helps you building datasets for phylogenetic analysis. *BMC Evolutionary Biology*.

[115] Dereeper A, Guignon V, Blanc G (2008). Phylogeny.fr: robust phylogenetic analysis for the non-specialist. *Nucleic Acids Research*.

[116] Castresana J (2000). Selection of conserved blocks from multiple alignments for their use in phylogenetic analysis. *Molecular Biology and Evolution*.

[117] Tamura K (1992). Estimation of the number of nucleotide substitutions when there are strong transition-transversion and G+C-content biases. *Molecular Biology and Evolution*.

[118] Huelsenbeck JP, Ronquist F (2001). MRBAYES: bayesian inference of phylogenetic trees. *Bioinformatics*.

[119] Ronquist F, Huelsenbeck JP (2003). MrBayes 3: bayesian phylogenetic inference under mixed models. *Bioinformatics*.

[120] Nylander JAA (2004). *MrModeltest v2. Program Distributed by the Author*.

[121] Legendre P, Makarenkov V (2002). Reconstruction of biogeographic and evolutionary networks using reticulograms. *Systematic Biology*.

[122] Makarenkov V (2001). T-REX: reconstructing and visualizing phylogenetic trees and reticulation networks. *Bioinformatics*.

[123] Makarenkov V (1999). An algorithm for the fitting of a tree metric according to a weighted least-squares criterion. *Journal of Classification*.

[124] Rocha-Olivares A, Fleeger JW, Foltz DW (2001). Decoupling of molecular and morphological evolution in deep lineages of a meiobenthic harpacticoid copepod. *Molecular Biology and Evolution*.

[125] Frost BW, Bucklin A (2009). Morphological and molecular phylogenetic analysis of evolutionary lineages within *Clausocalanus* (Copepoda: Calanoida). *Journal of Crustacean Biology*.

[126] Heath TA, Hedtke SM, Hillis DM (2008). Taxon sampling and the accuracy of phylogenetic analyses. *Journal of Systematics and Evolution*.

[127] Bergsten J (2005). A review of long-branch attraction. *Cladistics*.

