# Supplementary material for: Phylogenetic Information Content of Copepoda Ribosomal DNA Repeat Units: ITS1 and ITS2 Impact
Source: Biomed Res Int. 2014 Aug 18;2014:926342. doi: 10.1155/2014/926342 (PMC4151598; doi:10.1155/2014/926342)
Supplement: Supplementary file 1 — The Supplementary Material contains figures of substitution saturation plots of 28S, ITS1, ITS2, which allow to estimate the quality of phylogenetic signals; and ITS1 and ITS2 alignments of 8 species built in Mafft v.7 and treated by Gblocks v.0.91b, which give notion about sequences variability. [file 926342.f1.pdf]

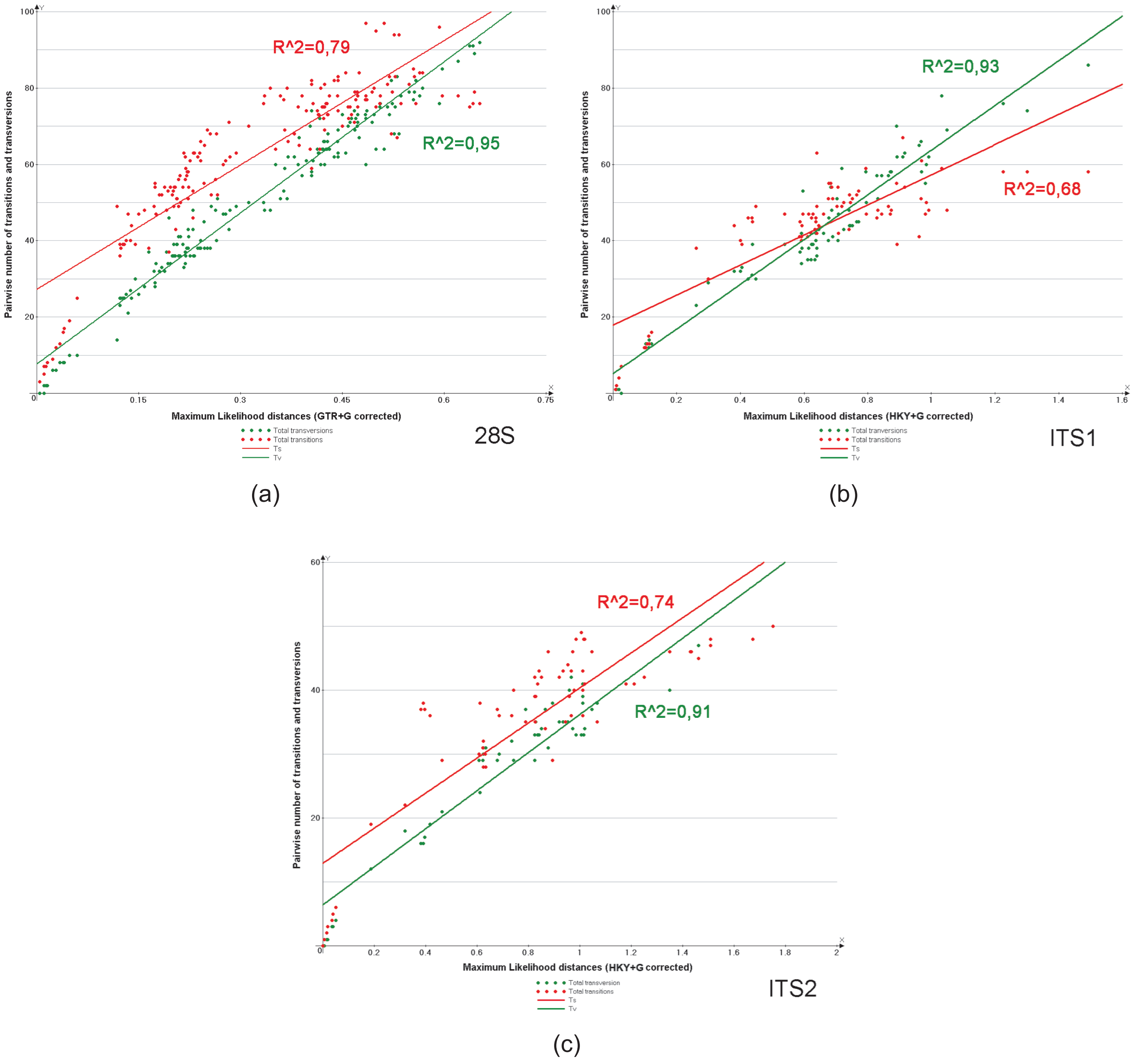

Supplementary figure 1. Substitution saturation plots of 28S, ITS1, ITS2.

Saturation plots of transitions (red) and transversions (green) calculated through pairwise sequence comparisons. Maximum-likelihood distances calculated using the substitution model that best fit the data are plotted against the number of substitutions at all nucleotide positions. Each point corresponds to the number of substitutions per pairwise distance value. Linear regression analysis of 28S gene fragment, ITS1 and ITS2 saturation graphs for transitions (red) and transversions (green); R^2, coefficient of determination (red, transitions; green transversions). (a) – 28S saturation plot; (b) – ITS1 saturation plot; (c) – ITS2 saturation plot.

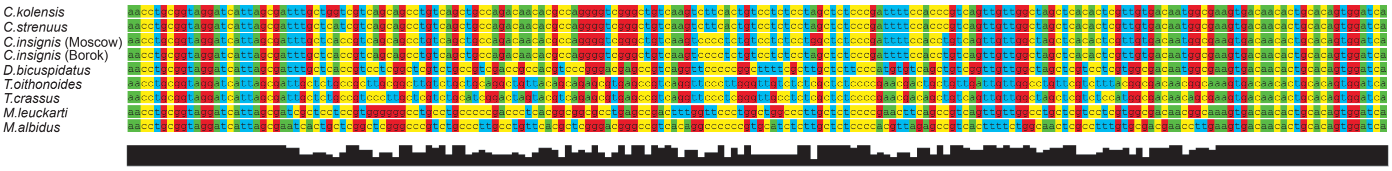

>*C.kolensis*
aacctgcggtaggatcattagcgatttgctggtcgtcagcagcctgtcagctgccagacaacacgccaggggtcgggctgtcaagtcttcactgtcctctcctagctctcccgattttccacccgtcagttgttggctagctcacactcgttgtgacaatggcgaagtgacaacactgcacagtggatca

>*C.strenuus*
aacctgcggtaggatcattagcgatttgctcatcgtcagcagcctgtcagctgccagacaacacgccaggggtcgggctgtcaagtcttcactgtcctctcctagctctcccgattttccacccgtcagttgttggctagctcacactcgttgtgacaatggcgaagtgacaacactgcacagtggatca

>*C.insignis* (Moscow)
aacctgcggtaggatcattagcgatttgctcaccgtcagcagcctgtcagctgccagacaacacgccaggggtcgggctgtcaagtcccctctgtcctctcctggctctcccgattttccacctgtcagttgttggctagctcacactcgttgtgacaatggcgaagtgacaacactgcacagtggatca

>*C.insignis* (Borok)
aacctgcggtaggatcattagcgatttgctcaccgtcagcagcctgtcagctgccagacaacacgccaggggtcgggctgtcaagtcccctctgtcctctcctagctctcccgattttccacctgtcagttgttggctagctcacactcgttgtgacaatggcgaagtgacaacactgcacagtggatca

>*D.bicuspidatus*
aacctgcggtaggatcattagcgatttgctcaccgtcctcggctcgtctgccgtcgaccgccacgtcccgggacgagccgtcaggttccccggcttttcgcttgctcttcccatgtgtcagctgtcggttgttggctagctcgtcctcgtggcgacaatggcgaagtgacaacactgcacagtggatca

>*T.oithonoides*
aacctgcggtaggatcattagcgattgctctgccgcttgcggcttgtctgctgcaggctgttacagcagagcgtgagccgtcaggttcccttgggttgtctctcgctctccccgaacgactgctgttgattgttggcctgttcgtctttacggcgacaacggcaaagtgacaacactgcacagtggatca

>*T.crassus*
aacctgcggtaggatcattagcgattgctctgccgtcccttgctcgtctgcatcggactagtacgtcagagcgtgagccgtcaggttccctcgggttgcctctcgctctccccgaacgacagctgtcagttgttggctagctcgtctccatggcgacaacagcgaagtgacaacactgcacagtggatca

>*M.leuckarti*
aacctgcggtaggatcattagcgatcgctcctccgtgggggcctgcctgcccccgaccctcacggcggcgcctgagccgactttggttccctggctggcccttgctctccccgaacttcagccgtcagttgttggcctgctcgtcctcgtggcgacaacggcaaagtgacaacactgcacagtggatca

>*M.albidus*
aacctgcggtaggatcattagcgaatcactgctcggctcgggcccgtctgcccttgcctgttcacgctcgggacgggccgtcacaggccccccgtgcatctcttgctctccccacgttagagccgtcacttttctggcaactcgcctttgtgcgacgaaccttgaagtgacaacactgcacagtggatca

(a)

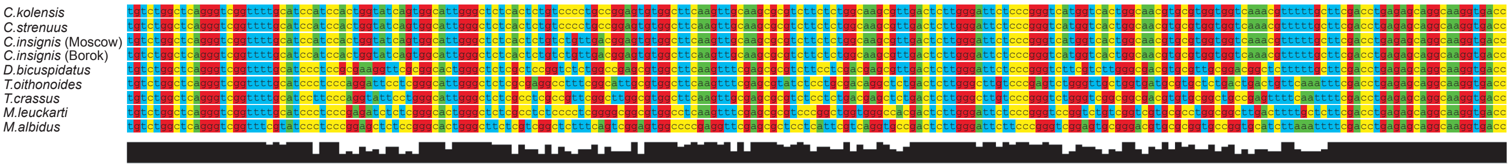

>*C.kolensis*
tgtctggctcagggtcggttttgcatccatccactggtatcagtggcattgggctctcactctgtcccctgccggagtgtggcttcaagttgcaagcgcgtcttctctggcaagcgttgactcttgggattctcccgggtcatggtcactggcaacgtgcgtggtggtcaaacgtttttgcttcgacctgagagcaggcaaggtgacc

>*C.strenuus*
tgtctggctcagggtcggttttgcatccatccactggtatcagtggcattgggctctcactctgtcccctgccggagtgtggcttcaagttgcaagcgcgtcttctctggcaagcgttgactcttgggattctcccgggtcatggtcactggcaacgtgcgtggtggtcaaacgtttttgcttcgacctgagagcaggcaaggtgacc

>*C.insignis* (Moscow)
tgtctggctcagggtcggttttgcatccatccactggtatcagtggcattgggctctcactctgtctgttgacggagtgtggcttcaagttgcaagcgcgtcttctctggcaagcgttgactcttgggattctcccgggtcatggtcactggcaacgtgcgtggtggtcaaacgtttttgcttcgacctgagagcaggcaaggtgacc

>*C.insignis* (Borok)
Tgtctggctcagggtcggttttgcatccatccactggtatcagtggcattgggctctcactctgtctgttgacggagtgtggcttcaagttgcaagcgcgtcttctctggcaagcgttgactcttgggattctcccgggtcatggtcactggcaacgtgcgtggtggtcaaacgtttttgcttcgacctgagagcaggcaaggtgacc

>*D.bicuspidatus*
tgtctggctcagggtcggttttgcatccctccgcgaaggttcgcggcactgggctctcgctccggtctctggccgagcgtggcttcaagtttcgagcgcgtcttcctcgacgagcgttgactcttgggattctcccgggtcttcgtcttgggcgacgtgcgttgcggacggctctttttgcttcgacctgagagcaggcaaggtgacc

>*T.oithonoides*
Tgtctggctcagggtcggttttgcatccctcccaggattcctcgggcattgggctctcgcgaggcctttcggcattgcgtggcttcaagtttcgagcgtatctcctgcgacaggctctgactcttgggcttgtcccgagtctgggttgctggtgatgcgtgctctgactgactgttcaaatttcgacctgagagcaggcaaggtgacc

>*T.crassus*
Tgtctggctcagggtcggttttgcatccttcccaggtattcctgggcattgggctctcgcctcgccgttcggcttggcgtggcttcaagttgcgagcgcgtctcctctgacgagctctgactcttgggcttgtcccgggtctgggtcggcggcgacgtgtgcggctgccgagttttcaatttcgacctgagagcaggcaaggtgacc

>*M.leuckarti*
tgtctggctcagggtcggttttgcatccctcccgagatctctcgggcactgggctctcgcctctcccctcggggcggcgtggcctcaagtttcgagcgcgtcccggctggtgggccacgactcttgggattctcccgggtccggtctgtcggtcgtgcgcctggcggcttgacttttgctcttcgacctgagagcaggcaaggtgacc

>*M.albidus*
tgtctggctcagggtcggtttcgtatccctcccggagctctccgggcactgggcttctcgtcggctctttcagtcggagtggccccgaggttcgagcgctcctcattcgtcaggtgccgactcttgggattcttcccgggtcggagtgcgggacgtgcgcggtgccggtgcatcttaaatttcgacctgagagcaggcaaggtgacc

(b)

Supplementary figure 2. ITS1 and ITS2 alignments of 8 species built in Mafft v.7 and treated by Gblocks v.0.91b.
(a) - ITS1 alignment (190 bp), (b) - ITS2 alignment (208 bp). The following parameters were used: for Mafft – G-INS-I with scoring matrix – 200PAM / k=2, gap opening penalty – 1.53 and offset value – 0.0; for Gblocks – minimum number of sequences for a conserved position – 6, minimum number of sequences for a flanking position – 9, maximum number of contiguous nonconserved positions – 8, minimum length of a block – 10, allowed gap positions – none.
